# Decline in Soil Microbial Abundance When Camelina Introduced Into a Monoculture Wheat System

**DOI:** 10.3389/fmicb.2020.571178

**Published:** 2020-11-19

**Authors:** Jeremy C. Hansen, William F. Schillinger, Tarah S. Sullivan, Timothy C. Paulitz

**Affiliations:** ^1^Northwest Sustainable Agroecosystems Research Unit, USDA-Agricultural Research Service, Washington State University, Pullman, WA, United States; ^2^Department of Crop and Soil Sciences, Washington State University, Pullman, WA, United States; ^3^Wheat Health, Genetics, and Quality Research, USDA-Agricultural Research Service, Washington State University, Pullman, WA, United States

**Keywords:** Camelina, crop rotation, glucosinolates, phospholipid fatty acid analysis, fallow, wheat, microbial community

## Abstract

Camelina [*Camelina sativa* (L.) Crantz] of the Brassicaceae family is a potential alternative and oilseed biofuel crop for wheat (*Triticum aestivum* L.)-based cropping systems of the Inland Pacific Northwest (PNW) of the United States. We investigated the effect of this relatively new rotational crop on soil microbial communities. An 8-year cropping systems experiment was initiated in 2007 at Lind, WA, to compare a 3-year rotation of winter wheat (WW)-camelina (C)-fallow (F) to the typical 2-year WW-F rotation. All phases of both rotations (total = 20 plots) were present every year to allow valid statistical analysis and data interpretations. Monoculture WW-F is the dominant system practiced by the vast majority of farmers on 1.56 million ha of cropland in the PNW drylands that receive <300 mm average annual precipitation. Microbial abundance and community composition were determined using phospholipid fatty acid analysis (PLFA) from soil samples collected during 3 consecutive years beginning in 2010. The abundance of fungi, mycorrhizae, Gram positive and negative bacteria, and total microbial abundance all declined over the 3-year period in the WW-C-F rotation compared to the WW-F rotation. All microbial lipid biomarkers were significantly less in fallow compared to WW of the WW-C-F rotation. The 2-year WW-F rotation demonstrated few differences in microbial lipid abundance and community structure between the rotation phases. Microbial abundance declined and community structure shifted in the 3-year WW-C-F rotation likely due to the combination of a *Brassica* crop followed by a 13-month-long fallow. The results of this study suggest that camelina in combination with a fallow period may disrupt microbial communities that have become stable under historical WW-F monocropping. Such disturbances have the potential to affect soil processes that have been provided by wheat-adapted microbial communities. However, the disruption appears to be short-lived with the microbial abundance of WW in the WW-C-F rotation, returning to similar levels observed in the WW-F rotation.

## Introduction

The productivity of semi-arid, cereal-based agroecosystems is most often limited by water and nutrients (Maaz et al., 2018). Farmers in semi-arid rainfed regions around the world encounter the challenge of producing economically-viable crops with variable and limited precipitation. Semi-arid areas of the world are characterized by times of drought accompanied by sporadic periods of precipitation (Shan, 2002). Farmers use soil and residue management practices that capture precipitation during the wet periods, maintain moisture in the soil, and then sow crops that efficiently use available water (Deng et al., 2005). Such practices are particularly important in the low-precipitation zone of the Inland Pacific Northwest (PNW) of the United States, where the 2-year winter wheat-fallow (WW-F) rotation, which produces only one crop every other year, is dominant as it provides greater yield stability, less economic risk, and higher net economic returns compared to alternative spring-sown cereal and broadleaf crops such as wheat, barley (*Hordeum vulgare* L.), oat (*Avena sativa* L.), pea; *Pisum sativum* L.), canola (WC; *Brassica napus* L.), condiment mustard (*Brassica* spp.), chickpea (*Cicer arietinum* L.), lentil (*Lens culinaris* L.), safflower (*Carthamus tinctorius* L.), sunflower (*Helianthus annuus* L.), and flax (*Linum usitatissimum* L.) so far tested (Schillinger et al., 2006; Young et al., 2014).

While WW-F cropping has been successful in the PNW drylands, crop diversification is widely known to increase agroecosystem resilience (Moonen and Barberi, 2008; Lin, 2011) and often provides economic benefits to farmers (Entz et al., 2002; Zentner et al., 2004). Crop diversification positively influences the composition and abundance of soil microorganisms (Matson et al., 1997; Dias et al., 2015) and interrupts pest and disease cycles that negatively impact wheat (Kirkegaard et al., 2008). Benefits of crop rotation in wheat-based rotations have been demonstrated in the United States (Schillinger et al., 2006), Australia (Anderson et al., 2005), Canada (Johnston et al., 2005), and many other wheat regions around the world. Large economic benefits from improved yields of wheat following *Brassica* crops have been reported in Australia (Smith et al., 2004; Angus et al., 2011). Such rotational benefits are often attributed to control of soil-borne diseases and weeds (Kirkegaard et al., 2000; Morra and Kirkegaard, 2002).

Camelina is a relatively new *Brassica* crop to the PNW and has received much less attention than canola. Worldwide interest in camelina has increased dramatically in the past 15 years due to its low input requirements, tolerance of abiotic stresses, unique edible oil and seed meal properties, and as feedstock for low-carbon-emission jet fuel (Berti et al., 2016). Camelina has shown potential in PNW cereal-based cropping systems (Guy et al., 2014). Camelina has better cold, heat, and drought tolerance and is less susceptible to disease and insects than canola (*Brassica napus* L.; Hulbert et al., 2012; Guy et al., 2014). There are reports from high-precipitation regions such as Minnesota, United States that up to 80% of camelina root mass can sometimes be found in the surface 30 cm of soil (Gesch and Johnson, 2015). However, numerous studies from other regions of the world show that camelina roots grow and extract soil water to a depth of 150 cm or greater (Berti et al., 2016; George et al., 2018; Schillinger, 2019), similar to that of wheat.

Camelina in a 3-year rotation of WW-C-F has been proposed as a potential alternative to the traditional WW-F rotation in the low-precipitation zone of the PNW (Hulbert et al., 2012; Long et al., 2016). Guy and Gareau (1998) reported that wheat grown after five different broadleaf crops in the 450–600-mm average annual precipitation zone of the PNW produced an average of 29% greater yield than wheat following wheat. However, increased yields are not always observed in wheat following camelina. Schillinger (2019) conducted an 8-year cropping systems study at Lind, WA to compare a 3-year WW-C-F rotation to the widely practiced 2-year WW-F system. Camelina seed yield ranged from 339 to 1,175 kg/ha and averaged 643 kg/ha. Winter wheat grain yield of 2,692 kg/ha in the 3-year rotation was significantly lower compared to 2,862 kg/ha in the 2-year WW-F system. Water content in the 180-cm soil profile was significantly lower (*p* < 0.001) after harvest of camelina compared to after WW harvest in the 2-year rotation. This soil water reduction was consistently measured throughout the ensuing 13-month fallow cycle and was likely the main factor for the WW grain yield differences in the two systems (Schillinger, 2019). Further agronomic data from the 8-year study are reported by Schillinger (2019). For these reasons, regional farmers have shown little interest in growing camelina.

Somewhat similar to the camelina agronomy findings reported by Schillinger (2019), in a 6-year on-farm rotation study conducted near Davenport, WA, Schillinger and Paulitz (2018) reported grain yield for spring wheat (SW) after winter canola (WC) was significantly reduced by an average of 17% compared with SW after WW. Measurements of profile soil moisture, soil nutrients, foliar and roots diseases, weeds, and root lesion nematodes were unable to explain the yield reduction (Schillinger and Paulitz, 2018). Further analysis of soil samples from this 6-year study at Davenport revealed a significant reduction in the abundance of fungi, mycorrhizae, and total microbial biomass in WC compared to WW (Hansen et al., 2019). Much like canola, camelina contains glucosinolates (GSLs), upon cell rupture during the decay of residue, which hydrolyze to produce isothiocyanates. Dimethyl-disulfide is another compound associated with the roots of camelina (Walsh et al., 2014). Production of isothiocyanates and dimethyl-disulfide contribute to the “biofumigation effect,” which can reduce the inoculum of soil-borne pathogens. However, the non-selectivity of these compounds has potential to also impact beneficial soil organisms.

We conducted a 3-year study in the aforementioned camelina cropping systems experiment at Lind, WA to determine the influence of camelina on soil microbial communities. Beginning in 2010, we collected soil cores for 3 consecutive years from the experiment. As the field experiment was established in 2007, a complete cycle of the 3-year WW-C-F had been completed and was in “full rotation” by the time of our first soil sampling. The objective of our study was to compare soil microbial communities associated with WW, C (camelina), and fallow in a 3-year rotation to those in the monoculture 2-year WW-F system. We hypothesized that the microbial communities of camelina would be differentially influenced by possible exposure to GSLs in the camelina rooting system, with reduced microbial abundance and shifts in the soil microbial community composition compared to wheat.

## Materials and Methods

### Site Description and Experimental Design

A long-term cropping systems experiment was initiated in 2007 at the Washington State University Dryland Research Station near Lind, WA, to compare a 3-year rotation of WW-C-F to the standard 2-year WW-F rotation. The soil is a Shano silt loam (coarse-silty, mixed, superactive, and mesic Xeric Haploxeroll) with 0–2% slope and depth greater than 2 m to basalt bedrock (NRCS, 2018). These soils are composed of 10% clay, 56% silt, and 34% sand. Organic matter content in the surface of 15 cm of soil is 0.7% and soil pH is 6.4. Soil texture is uniform throughout the profile. Annual precipitation at the site averages 244 mm. In this Mediterranean-like climate, most precipitation occurs during late fall and winter and diminishes from April to June. The months of July to September are mostly dry.

Experimental design was a randomized complete block with four replications. All phases of both rotations were present every year for a total of 20 plots (i.e., 8 plots for WW-F and 12 plots for WW-C-F). Individual plot size was 9 m × 76 m and the experiment covered 1.39 ha. The land used for the experiment was in WW production in 2006. To initiate the experiment in 2007, 8 plots were left fallow, 4 plots were planted to camelina, and 8 plots planted to spring wheat. The planting of spring wheat in 2007 was needed to provide 8 plots for fallow the next year. This temporal staggering or “setup” allowed for the proper establishment of both the WW-F and WW-C-F rotations in all 20 plots in 2008 and a full rotation cycle (i.e., including fallow) to be completed in the 3-year WW-C-F system in 2009. Such proper rotation setup is required for reporting in long-term cropping systems experiments (Cady, 1991). Thus, 2010 was the first of 3 consecutive years for data collected, analyzed, and reported in this paper. All data presented here are from soil samples obtained from the field experiment in mid-May of 2010, 2011, and 2012.

### Field Operations

In both rotations, conservation tillage practices were used during the 13-month-long fallow phase. Stubble from the previous crop was left standing and undisturbed from harvest in July through the winter. Glyphosate herbicide was applied in March to control weeds. Primary spring tillage plus fertilizer injection with an undercutter implement was conducted at a depth of 13 cm in late April or early May to sever soil capillary pores and channels to retard the evaporation of soil water during the hot, dry summer months. One or two field operations with a rodweeder (also a noninversion implement) were conducted at a depth of 10 cm in late spring and/or July to control weeds. The fallow management practices used in the study are considered among the best management practices for WW production and control of wind erosion in the low-precipitation region of the PNW (Papendick, 2004). Winter wheat was planted into fallow with a deep-furrow drill in early September. Fallow in both 2- and 3-year rotations received the same type and timing of field operations.

In the 3-year rotation, camelina was direct seeded into the standing and undisturbed stubble of the preceding WW crop in late February or early March with a no-till hoe drill equipped with paired-row seed openers. Glyphosate herbicide was applied 7–10 days prior to planting camelina to control volunteer wheat and other weeds. Fertilizer was stream jetted on the soil surface after camelina seedling emergence. Detailed descriptions of all field operations conducted throughout the experiment are described by Schillinger (2019). Management guidelines for camelina production in the PNW, such as used in this study, have been reported by Hulbert et al. (2012).

### Soil Sampling

Soil for microbial analyses was collected from 0 to 5, 5 to 10, and 10 to 15 cm depths from all 20 plots in mid-May, when WW was in the stem elongation phase and camelina was at early inflorescence. From each replicated plot, eight 3.0-cm diameter soil cores at each depth were combined to form a composite sample. Of the eight cores, four cores were collected within the crop row and four cores were collected between crop rows across the length and width of the plot, avoiding borders. Sample collection occurred each year at the same time from 2009 to 2012. Samples were immediately transported in a cooler, on ice, in the dark to the laboratory located in Pullman, WA. Subsamples of the composite soil were collected in sterile tubes and stored at −80°C until analysis.

### Soil Chemical Analyses

Soil pH and electrical conductivity (EC) were determined by preparing a slurry of 1:1 soil to distilled, deionized water (McLean, 1982). Centrifuge tubes containing the soil slurry were mixed end-over-end overnight at room temperature. The slurry was then centrifuged at 4000 rpm to separate solid from liquid. The pH of the soil solution was determined with an Orion Research 811 (Boston, MA) pH meter, and EC was measured using a digital conductivity meter (VWR International, Bristol, CT).

### Soil Microbial Enzyme Activity

The *β*-glucosidase (B-glu) and dehydrogenase activities (DEA) were determined (<2 mm sieved) as an indicator of microbial activity as described in Tabatabai and Dick (2002). The colorimetric measurement was completed on a BioTek microplate reader (BioTek, Winooski, VT), set to a wavelength of 410 nm and 492 nm for B-glu and DEA, respectively. Results for B-glu are expressed in *μ*g of *p*-nitrophenol (PNP) released per gram of dry soil per hour and μg of 2,3,5-triphenyl formazan (TPF) per gram of dry soil per hour for DEA. Calculation of PNP formation was accomplished by reference to a calibration curve prepared with known concentrations of PNP ranging from 10 to 50 μg PNP 100 ml^−1^. Calculation of TPF formation was achieved by reference to a calibration curve prepared with known concentrations of TPF ranging from 500 to 2000 μg TPF 100 ml^−1^.

### Soil Microbial Abundance and Community Composition

The protocol used for whole-soil phospholipid fatty acid (PLFA) extraction is described by Buyer et al. (2010). Procedures generally followed Bligh and Dyer (1959) as described by Petersen and Klug (1994) and modified by Ibekwe and Kennedy (1998). The standard protocol used for PLFA analysis in this study has been described in detail by Hansen et al. (2019). A gas chromatograph (Agilent Technologies GC 6890, Palo Alto, CA) equipped with a fused silica column and flame ionizer detector was used to analyze phospholipids from the PLFA extractions. Integration and analysis of samples were conducted by ChemStation software (Agilent Technologies GC 6890, Palo Alto, CA). Microbial Identification Systems, Inc. (Newark, DE) software provided parameters that were used for peak identification and integration of areas. Peak chromatographic responses were converted to mol responses by reference to an internal standard (methyl nonadecanoate fatty acid 19:0) that was added before the methylation step. The MIDI Eukaryote method and fatty acid library were used, with the split ratio changed from 1:100 to 1:50 to increase sensitivity. Peak areas of carbon chain lengths between 12:0 and 20:0 were summed into biomarker groups as described by Hansen et al. (2018). Gram-positive (Gram+) bacteria are represented by the sum of iso and anteiso branched fatty acids (Zelles, 1999). Monounsaturated fatty acids and cyclopropyl 17:0 and 19:0 were used as biomarkers for Gram-negative (Gram−) bacteria (Zelles, 1997). Fungi were identified by fatty acids 18:2 *ω*6c and 18:1 ω9 (Vestal and White, 1989; Frostegård and Bååth, 1996; Bååth, 2003). Fatty acids 16:1 ω5c (Olsson, 1999) and 20:1 ω9c (Madan et al., 2002) were used to quantify arbuscular mycorrhizae (AM fungi). Total of all PLFA (T-PLFA) was considered to represent viable microbial abundance. Bacterial to fungal ratios (B to F ratio) were calculated from the total of AM fungi, fungi, and Gram+ and Gram– bacteria.

### Statistical Analysis

ANOVA was conducted for soil enzyme activity and microbial biomarker data using a randomized complete block design ANOVA with treatment as the fixed-effect factor and year as the random effect factor. There was not a significant time × treatment interaction, so data from years 2010 to 2012 were reported as means across years. Data were assessed through linear mixed models (PROC MIXED SAS version 9.4; SAS Institute, 1999), to determine differences in soil properties, enzyme activities, and microbial group abundance. Comparisons included means for crop/fallow irrespective of rotation and means for crop/fallow phase of each rotation. This was done because microbial communities vary with different crops in a rotation sequence (Grayston et al., 1998; O’Donnell et al., 2001) and patterns are frequently found to be associated with plant species (Marschner et al., 2001; Ladygina and Hedlund, 2010). Analyzing the data this way can help to distinguish the influence of the current crop/fallow from the cumulative influence of the rotation and vice versa. Data that are discussed for crop/fallow phase irrespective of rotation are hereafter referred to as crop/fallow, while the crop/fallow phase including rotation will be referred to as crop/fallow phase of each rotation (2-year or 3-year). Mean comparisons to identify significant differences were performed at *p* ≤ 0.05. Where *p* was significant, Fisher’s protected LSD was used to estimate differences between treatment means.

To identify the influence treatments had on microbial community composition, discriminant function analysis (DFA) was performed on biomarker groups. Previous to DFA, peak chromatographic responses for carbon chain lengthen between 12:0 and 20:0 were summed into biomarker groups. Discriminant function analysis identified the linear combination of variables (CVs, canonical variates) that best separated microbial community structure associated with experimental treatments (Dangi et al., 2012). We used DFA (Buyer et al., 2002; Dangi et al., 2012) to compare soil microbial community structure for each crop/fallow phase and crop/fallow phase by rotation. Studies that aim to characterize the microbial community by PLFA use DFA to explore the underlying effect of soil type (Buyer et al., 1999), fertilizer (Lazcano et al., 2013), and plant-derived allelochemicals on microbial community structure. CVs generated by this analysis identified the linear combination of variables that best separated the samples by crop/fallow phase and rotation. This multivariate eigenvalue technique maximizes the among-group variation relative to the within-group variation, which allows for distinction between previously established groups (Lazcano et al., 2013). Bi-plots of the first two canonical variables were graphed to summarize group differences (Buyer et al., 2002). For clarity, the group means are shown and not individual points (*n* = 60) and are accompanied by a mean ellipse at the 95% CI. Two groups are considered significantly different when the mean ellipses do not intersect (JMP, n.d., p. 1989–2007). The bi-plot vectors represent covariates (biomarker groups) with the length and direction of each vector proportional to the degree of association with the first two CVs. For all bi-plots presented in this paper, the first CV accounts for the bulk of the variance. Therefore, separations along the horizontal axis are more significant than those occurring along the vertical axis. Discriminant function analysis was performed using the JMP software program (JMP, SAS Institute, 1999) at the *p* ≤ 0.05 significance level.

## Results

### Soil Properties and Enzyme Activities

**Figure 1:**
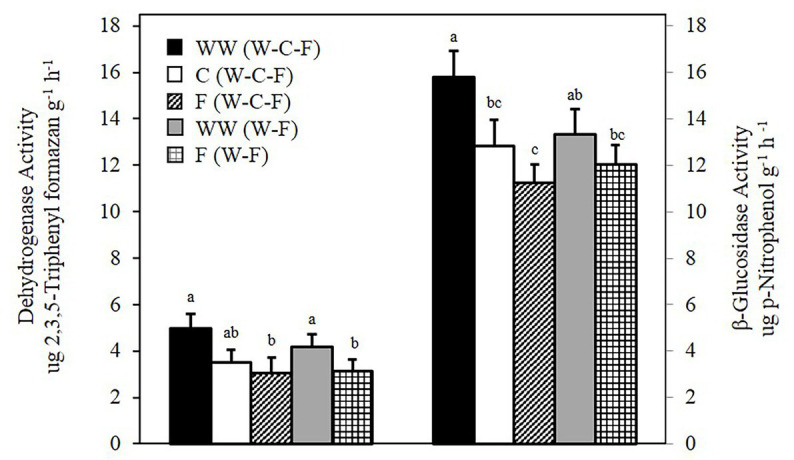
Dehydrogenase and β-glucosidase activity of winter wheat (WW), camelina (C), and fallow (F). Values are least square means across crop years 2010–2012. Error bars represent standard error. Values among each assay with different letters are significantly different (*p* < 0.05).

At the 0–5 cm depth in the 3-year rotation (WW-C-F), DEA decreased from WW > C > F, with WW significantly greater than fallow (Figure 1). In the 2-year (WW-F) rotation, DEA in WW was significantly greater than in fallow (Figure 1). Activity of B-glu followed a similar pattern as DEA, decreasing from WW > C > F with WW significantly greater than F. In the 2-year rotation, B-glu activity of WW and F were not significantly different (Figure 1). Significant differences were not observed for either enzyme assay at the lower depths in either rotation (data not shown).

There were no significant differences among crop and fallow phases of either rotation for pH or EC at the 0–5 cm depth. Values of pH and EC were 6.3, 6.2, 6.1, 6.4, and 6.3 and 278, 242, 235, 263 and 229 *μ*S cm^−1^ for WW (WW-C-F), C (WW-C-F), F (WW-C-F), WW (WW-F), and F (WW-F), respectively. Similarly, there were also no differences for pH and EC at the lower sampling depths (data not shown).

### Soil Microbial Abundance and Community Composition

**Figure 2:**
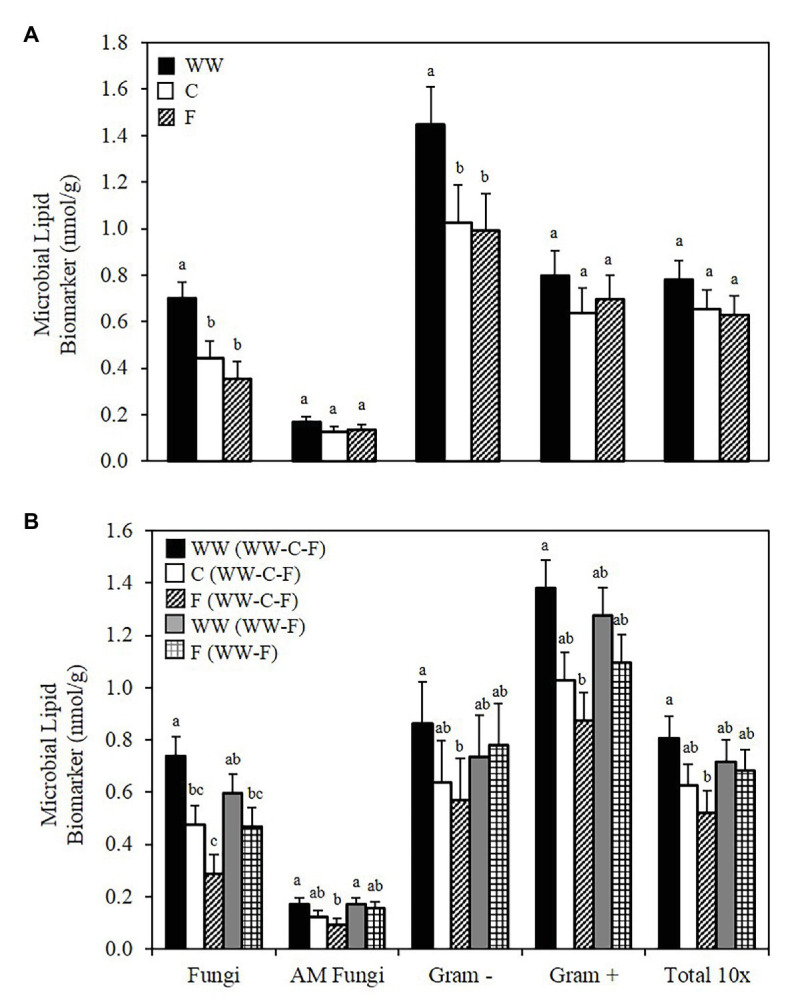
Soil microbial lipid abundance. Biomarker groups and total PLFA (T-PLFA) concentrations (nmol/g) of soil. Values are least square means across crop years 2010–2012. **(A)** Shows the average across both WW-C-F and WW-F rotation systems. **(B)** Shows the average for each crop of the separate rotation systems. Error bars indicate standard error. Values within each biomarker group with different letters are significantly different (*p* ≤ 0.05). WW, winter wheat; C, camelina; F, fallow.

Combined biomarkers for bacteria demonstrate a significant decline from WW to C at all depths and a significant decline in the fungal biomarkers in two of the three depths (Table 1). Biomarker data for each crop/fallow show that WW had significantly greater abundance of fungi and Gram− bacteria compared to C and F (Figure 2A). No differences in AM fungi, Gram+ bacteria, and T-PLFA were observed among WW, C, or F (Figure 2A). However, when comparing the crop/fallow phase for both rotations, crop by rotation treatments displayed significant differences in all biomarker groups (Figure 2B). In the 3-year rotation, abundance of all biomarkers at the 0–5 cm depth decreased from WW > C > F with WW significantly greater than fallow in all biomarkers (Figure 2B). A similar trend of WW > C > F in the 3-year rotation was found in the 5–10 and 10–15 cm depths (Supplementary Table S1). In the 2-year rotation, there were no differences between the WW and F phases in the surface soil or lower depths (Supplementary Table S1). When comparing the WW phases of the two rotations, no significant differences in biomarkers for bacteria and fungi are observed (Table 1; Figure 2B). The same observation of non-significant differences is true when comparing the C and F phases of the 3-year rotation (Table 1; Figure 2B).

**Table 1:** Total bacteria, total fungi, and bacterial to fungal ratios (B to F) at 0–5, 5–10, and 10–15 cm depths from crop years 2010 to 2012.

Depth	Treatment (rotation)	Bacteria (nmol/g)	Fungi (nmol/g)	B to F Ratio
0–5 cm	WW (WW-C-F)	2.24 A	0.91 A	2.46 B
	C (WW-C-F)	1.66 B	0.60 BC	2.78 B
	F (WW-C-F)	1.44 B	0.38 C	3.77 A
	WW (WW-F)	2.01 A	0.77 AB	2.62 B
	F (WW-F)	1.87 AB	0.63 BC	2.99 B
5–10 cm	WW (WW-C-F)	1.85 A	0.54 A	3.43 AB
	C (WW-C-F)	1.28 B	0.37 AB	3.46 AB
	F (WW-C-F)	1.07 B	0.29 B	3.69 A
	WW (WW-F)	1.44 AB	0.46 AB	3.13 AB
	F (WW-F)	0.92 B	0.32 B	2.88 B
10–15 cm	WW (WW-C-F)	1.23 A	0.37 A	3.32 B
	C (WW-C-F)	0.74 B	0.22 B	3.36 B
	F (WW-C-F)	0.66 B	0.20 B	3.30 B
	WW (WW-F)	1.20 A	0.36 A	3.33 B
	F (WW-F)	0.98 AB	0.23 AB	4.26 A

Values are least square means across all years. Values within a column with different letters are significantly different (*p* ≤ 0.05).

The bacterial:fungal ratio was significantly greater in F compared to WW and C in the 3-year rotation at the 0–5 cm depth. No differences were observed between WW and F in the 2-year rotation at the 0–5 cm depth (Table 1). At the 5–10 cm depth, the bacterial:fungal ratio for fallow (WW-C-F) was greater compared to fallow (WW-F), but this was the only difference between ratios at that depth. At the 10–15 cm depth, the bacterial:fungal ratio for fallow (WW-F) was significantly greater than all other treatment combinations (Table 1). The treatments with a greater bacterial:fungal ratio experienced an increase, or maintained, greater bacterial abundance when compared to the fungal community.

**Figure 3:**
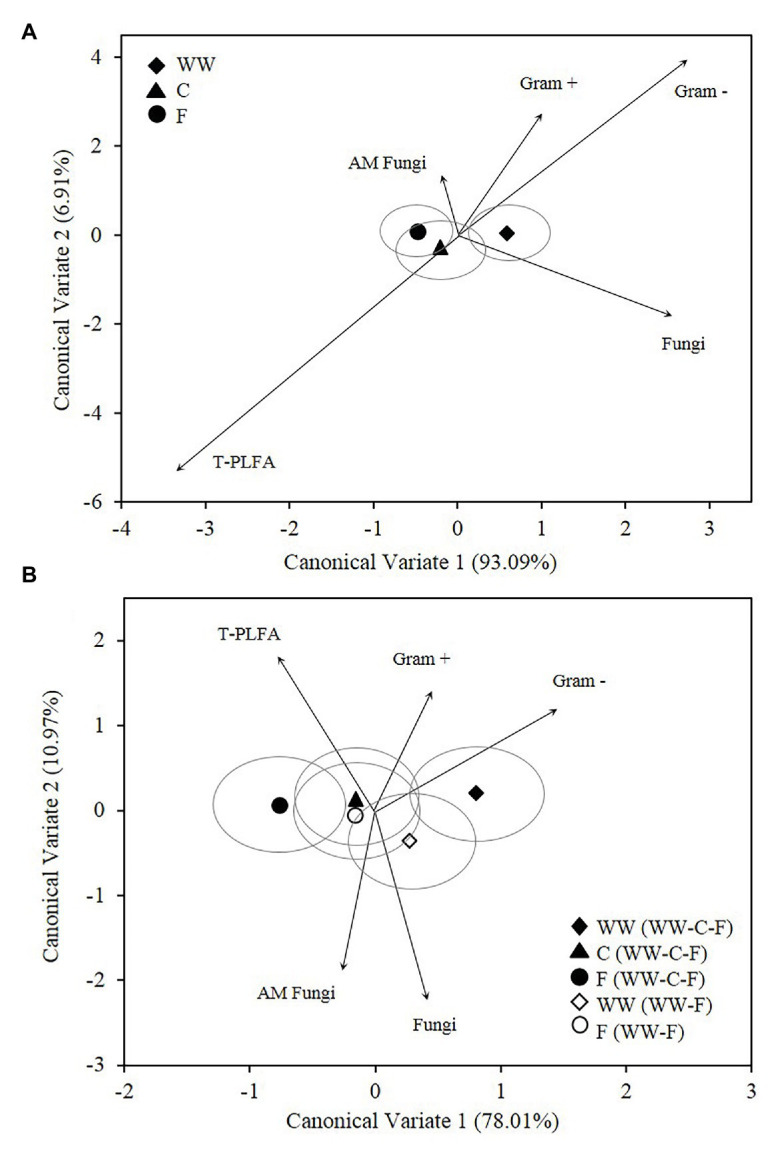
Canonical variates for lipid biomarker groups. Biomarker groups and total PLFA (T-PLFA) of soil from 2010 to 2012. Classification variables for the canonical analysis were crop/fallow **(A)** and crop/fallow phase of each rotation **(B)**. Vectors represent standardized canonical coefficients and indicate the contribution of each biomarker group to each canonical variate. Each point represents the group mean and is accompanied by a mean ellipse at the 95% CI (treatments groups that differ significantly have confidence ellipses that do not intersect). WW, winter wheat; C, camelina; F, fallow.

As with the abundance data, the microbial community structure data were analyzed for differences in crop/fallow treatment and crop/fallow phase of each rotation. Microbial biomarkers averaged across all years (2010–2012) are graphically represented as bi-plots for crop species and fallow (Figure 3A) and crop/fallow phase of each rotation (Figure 3B). Each classification variable is represented by the mean and is accompanied by a mean ellipse at the 95% CI. Soil microbial communities associated with crop/fallow treatment separated along the first CV, with WW positively correlated to CV1 (Figure 3A). Discriminant functions associated with CV1 and CV2, accounted for 93 and 6% of the variance, respectively, for a total explained variance of 99% (Figure 3A). Because CV1 accounts for the bulk of the variance, separations along the horizontal axis are more significant than those occurring along the vertical axis. The mean ellipses of two groups are significantly different if the ellipses do not intersect (JMP, SAS Institute, 1999). Accordingly, WW and F communities are considered significantly different, while the communities of C and F and WW and C are not. Differentiation between the microbial communities of WW and F within the DFA based on the amount and type of PLFAs was significant at *p* < 0.05 (Table 2). This demonstrates separation based on the presence or absence of a crop is greater than the separation between crop species. Bi-plot vectors represent covariates (biomarker groups) with the length and direction of each vector proportional to the degree of association with the first two canonical variates. The vectors of fungi, Gram+ bacteria, and Gram− bacteria are all positively correlated to CV1 with Gram+ and Gram− bacteria positively correlated to CV1 and CV2 (Figure 3A).

**Table 2:** Structure matrix (pooled within canonical structure) and biomarker means (group centroid) for soil samples collected from spring 2010 to spring 2012.

Classification variable	Crop			Crop by rotation	
Structure loading	CV1	CV2		CV1	CV2
Fungi	0.98	−0.04	Fungi	0.98	−0.04
AM fungi	0.56	−0.59	AM Fungi	0.56	−0.59
Gram− bacteria	0.53	−0.26	Gram− bacteria	0.53	−0.26
Gram+ bacteria	0.42	−0.01	Gram+ bacteria	0.42	−0.01
Total	0.53	−0.09	Total	0.53	−0.09
Group centroids					
Winter wheat	*0.59*	0.03	WW (WW-C-F)	*0.80*	0.20
Camelina	−0.21	−0.26	C (WW-C-F)	−0.16	0.14
Fallow	*−0.48*	−0.09	F (WW-C-F)	*−0.76*	0.07
			WW (WW-F)	0.27	−0.35
			F (WW-F)	−0.16	−0.05

Bold italics indicate treatments that differ significantly at the 95% confidence interval.

When analyzed as crop/fallow phase of each rotation, the only communities that were significantly different were those of WW and fallow in the 3-year rotation (WW-C-F; Figure 3B). These two communities separated along CV1, which accounts for 78% of the variance. Differentiation between the microbial communities of WW and fallow in the 3-year rotation within the discriminant function analysis is based on the amount and type of PLFAs and was significant at *p* < 0.05 (Table 2). While there is separation between WW and fallow (WW-F), the mean ellipses overlap and, thus, indicating that the two communities are not significantly different (Figure 3B). When observing the means and mean ellipses of camelina (WW-C-F) and F (WW-F), the community structure appears to be very similar (Figure 3B). The vectors of fungi, Gram+ bacteria and Gram− bacteria are all positively correlated to CV1 with Gram+ bacteria and Gram− bacteria positively correlated to both CV1 and CV2 (Figure 3B).

## Discussion

A key take-home agronomic message from the 8-year camelina cropping systems study at Lind, WA (i.e., the study for this paper) was that soil water content in the 180 cm soil profile was significantly lower following camelina in the 3-year WW-C-F rotation compared to after WW in the 2-year WW-F system (Schillinger, 2019). This was likely due to growing two crops (i.e., WW + C) back-to-back without a year of fallow in between. This soil water deficit persisted throughout the ensuing fallow year. Thus, there was less available water at time of planting WW in the WW-C-F system than there was for WW-F (Schillinger, 2019). In this current paper, we report decline in bacterial and fungal abundance during the camelina and fallow phases of the 3-year WW-C-F rotation and in the fallow phase of the 2-year WW-F system (Table 1), but found no such differences in microbial abundance in the two rotations for WW (Table 1; Figure 2B). Thus, the soil microbial communities in the two rotations were in synch during the WW phase of the two rotations, which lend further credence that the WW grain yield differences reported by Schillinger (2019) were mostly due to soil water.

Soil microbial enzyme activities have been reported to be influenced by changes in crop species and rotation sequence (Acosta-Martinez et al., 2007; Berg and Kornelia, 2009). Dehydrogenase enzymes play a significant role in the oxidation of soil organic matter and are considered good indicators of microbial activity (Garcia-Gil et al., 2000). Significant differences in DEA activity were observed between WW and fallow in both the 2- and 3-year rotations. The differences observed in B-glu were similar to DEA in the 3-year rotation with no differences seen in the 2-year rotation. In general, DEA and B-glu activity were greater in WW compared to the other treatments in their respective rotation. It has been demonstrated that cropped plots often have higher microbial activity compared to fallow (Drijber et al., 2000), and the presence of plants is a major driver of microbial community structure (Donn et al., 2015). This agrees with our results, which showed greater enzyme activity associated with cropped plots compared to fallow.

Changes in microbial abundance and community structure can be directly or indirectly affected by crop species, rotation sequence, and cropping intensity (Bünemann et al., 2008; Zhang et al., 2014). When analyzed by crop/fallow treatment, the abundance of fungi and Gram− bacteria in WW were significantly greater than in camelina or fallow, while AM fungi, Gram+ bacteria and T-PLFA were not different. However, when considering the crop/fallow phase of each rotation, the differences become more apparent. Significant reductions in biomarkers took place in the WW-C-F rotation. Reduction in fungi from WW to C was significant while the decrease from C to F was not significant. For all other biomarkers and T-PLFA, there was no significant decrease from WW to C. Still, the decrease in microbial lipid abundance from WW in the first year, to fallow in the third year, was significantly different. However, differences of enzyme activity and microbial abundance in the 2-year WW-F rotation were not observed. These results indicate that the microbial community structure of the WW-F rotation could be partially an artifact of the legacy crop effect (Zhang et al., 2014), because the historical (>100 years) farming practice in our field experiment and, indeed, throughout the low-precipitation cropping region of the PNW, has been almost exclusively a WW-F monoculture system (Schillinger et al., 2006).

Because camelina is a *Brassica* crop containing GSLs and volatile organic compounds, the decrease in soil fungal abundance in camelina following WW could be attributed to exposure to residues and root exudates of camelina. Related studies conducted in the same region demonstrated that *Brassica* crops included as rotational crops led to decreased microbial biomass in canola compared to WW in both the bulk (Hansen et al., 2019) and rhizosphere soil (Hansen et al., 2018) determined by PLFA analysis. Similarly, high-throughput sequencing of rhizosphere soil demonstrated that canola can disrupt microbial communities that have developed under a long history of wheat monocropping (Schlatter et al., 2019). A slow and steady release of 2-PEITC, the dominant canola root GSL (Kirkegaard et al., 2000; Marschner et al., 2003), is sufficient to shift the active portion of the soil microbial community composed of bacteria and fungi (Rumberger and Marschner, 2003; van Dam et al., 2009). Camelina contains three main GSLs identified as 9-methyl-sulfinyl-nonyl-GSL, 10-methylsulfinyl- decyl-GSL, and 11-methyl-sulfinyl-undecyl- GSL (Jiang et al., 2016). In addition to GSL, camelina root exudates contain the volatile sulfur containing compound dimethyl disulfide (Walsh et al., 2014). When compared to the GSL 2-PEITC, the camelina root exudate dimethyl disulfide alone can inhibit the nitrification process by 30% and by greater than 35% when combined with 2-PEITC (Bending and Lincoln, 2000).

When analyzed by crop/fallow, community structure as determined by DFA demonstrated significant dissimilarities based on the presence or absence of a crop, while the separation between crop species was not significant. This is supported by the results of Donn et al. (2015), who determined that in a wheat-fallow cropping system, the major driver of bacterial community structure in soil is the presence of plants. Positive correlation to CV1 suggests that Gram+ bacteria, Gram− bacteria, and fungi were responsible for the separation of WW from fallow. Likewise, bacterial diversity was greater and fungal diversity was either greater or unchanged in the wheat portion of another wheat-fallow study (Castro et al., 2016).

When including the crop/fallow phase of each rotation in the analysis, the community of WW (WW-C-F) is clearly discriminated from the fallow (WW-C-F) community. Positive correlation to both CVs suggests that Gram− bacteria and Gram+ bacteria were the largest contributors to discrimination. This separation among treatments along the first CV is similar to the pattern of significance observed in the microbial abundance data. In the 3-year rotation, a progressive decrease in microbial abundance resulted in a significant reduction across all biomarkers and a shift in the fallow community structure from that which was developed under long-term monoculture wheat in a WW-F rotation. This change in microbial community was most likely a combination of a fallow period preceded by a broadleaf break crop (Smith et al., 2004). In addition, camelina is a non-host for AM fungi (Johnston et al., 2005; Valetti et al., 2016) and contains GSLs with the potential to reduce microbial biomass (Rumbereger and Marschner, 2003; Hansen et al., 2019) and disrupt wheat monoculture adapted microbial communities (Schlatter et al., 2019). Soil organic matter content at our site was 0.7% in the surface (15 cm). In low organic matter soils, the absence of a plant and the labile carbon source it provides can influence the stability of the microbial community (Lu et al., 2002; Farrar et al., 2003; Ladygina and Hedlund, 2010). This becomes evident when comparing the WW phase of the two rotations to the fallow phase with the absence of plant roots or the camelina phases, which would have less root mass than the fibrous roots system of the WW phase.

An important observation is that the abundance and community structure of WW and fallow in the 2-year rotation were not significantly different. In contrast, significant differences in abundance and community structure of the 3-year rotation were observed and can be partially explained by previous work that reported greater diversity and abundance in a cropped treatment over fallow (Acosta-Martinez et al., 2007) or by reduced soil moisture following camelina harvest that persisted into the subsequent fallow period reported by Schillinger (2019). A recent study conducted in a Canadian semi-arid agroecosystem also reported that pH had a greater influence than crop, crop rotation or seasonal variation in controlling the structure of the bacterial community (Bainard et al., 2016). Soil pH could partially explain the similarities between WW and fallow in the 2-year rotation. Microbial biomass in our study was dominated by bacteria, at 2–3 times greater abundance than fungi. We also found no difference in pH between WW and fallow (6.4 and 6.3) and within the pH range for optimal bacterial growth that is near neutral (Matthies et al., 1997; Rousk et al., 2010). While no changes in pH may help to explain the lack of microbial community differences in the WW-F rotation, the same consistent pattern of pH was observed in the WW-C-F rotation, where differences in microbial abundance were observed. In addition to pH, the crop legacy effect likely contributed to the similarities in communities of the WW-F rotation. Microbial communities that remain under the historic farming practice of WW-F appear to be more stable and less influenced by WW or fallow phase and seasonal variation (Bainard et al., 2016). However, the communities of the 3-year rotation fluctuated in response to the addition of camelina to the rotation.

One of the goals of this study was to determine how the inclusion of camelina into historical WW-F rotations would affect the microbial community that has developed under this system. It then becomes important to compare the WW phase of both the rotations to determine if, after going through a complete rotation and returning to WW, the community experiences a decline or the previous levels of microbial biomass are restored. When comparing the abundance of fungi and bacteria associated with WW of the two rotations, we see that at all depths there was no difference in the WW phase between the WW-C-F and WW-F rotations (Table 1; Figure 2B). Though we see an overall decline in microbial biomarkers in the WW-C-F rotation, the microbial community demonstrates resilience through its ability to return to the levels observed in the WW-F rotation. This indicates that WW is able to recruit back a large microbial community from the previous community, whether there was a year of F before WW (as in WW-F) or back-to-back years of C plus F (as in WW-C-F).

## Conclusion

Results presented here show a sequential decline in microbial biomarkers associated with the 3-year WW-C-F rotation. This demonstrates how easily the microbial community can change in response to perturbations in a soil environment, in this case by introduction of a *Brassica* crop species and new crop rotation. The intermediate step between WW and fallow provided by camelina by itself is not enough to cause a significant effect. However, the combination of a camelina crop preceding a fallow period was enough to cause a significant decline in the microbial community. On the other hand, the lack of change between the WW and fallow phases in the 2-year rotation demonstrates the microbial community stability under the historic long-term farming practice. Furthermore, WW in the WW-C-F and WW-F rotations are also not significantly different. This indicates that even if communities are depleted under the C and F phase in the 3-year rotation, there are enough surviving members and residual inoculum that WW is able to recruit back a large microbial community and restore previous levels of microbial biomass.

## Data Availability Statement

The raw data supporting the conclusions of this article will be made available by the authors, without undue reservation.

## Author Contributions

WS, TP, and JH designed the experiment. JH performed all laboratory analysis, statistical analysis, evaluated the data, and drafted the manuscript. JH, WS, TP, and TS contributed to the final version of the manuscript.

### Conflict of Interest

The authors declare that the research was conducted in the absence of any commercial or financial relationships that could be construed as a potential conflict of interest.
