# Supplementary material for: Decline in Soil Microbial Abundance When Camelina Introduced Into a Monoculture Wheat System
Source: Front Microbiol. 2020 Nov 19;11:571178. doi: 10.3389/fmicb.2020.571178 (PMC7710528; doi:10.3389/fmicb.2020.571178)
Supplement: Supplementary file 1 [file Table_1.docx]

Supplementary Material

| **Table S1**. Absolute abundance (nmol/g) of microbial lipid groups at 0-5, 5-10, and 10-15 cm depths from crop years 2010 to 2012. Values are least square means (n=80) across all years. Means within a column at each depth with different letters are significantly different (*p* ≤ 0.05) | | | | | | |
| --- | --- | --- | --- | --- | --- | --- |
| Depth | Treatment (Rotation) | Fungi | AM Fungi | Gram- | Gram+ | Total |
| 0 to 5 cm | WW (WW-C-F) | 0.74 A | 0.17 A | 0.86 A | 1.38 A | 8.08 A |
|  | C (WW-C-F) | 0.47 BC | 0.13 AB | 0.64 AB | 1.03 AB | 6.25 AB |
|  | F (WW-C-F) | 0.29 C | 0.09 B | 0.57 B | 0.88 B | 5.22 B |
|  | WW (WW-F) | 0.59 AB | 0.17 A | 0.73 AB | 1.28 AB | 7.18 AB |
|  | F (WW-F) | 0.47 BC | 0.16 AB | 0.78 AB | 1.09 AB | 6.81 AB |
| 5 to 10 cm | WW (WW-C-F) | 0.42 A | 0.12 A | 0.71 A | 1.14 A | 6.73 A |
|  | C (WW-C-F) | 0.28 AB | 0.09 AB | 0.47 AB | 0.81 AB | 4.69 AB |
|  | F (WW-C-F) | 0.23 B | 0.06 B | 0.39 B | 0.68 B | 3.91 B |
|  | WW (WW-F) | 0.34 AB | 0.12 A | 0.53 AB | 0.91 AB | 5.39 AB |
|  | F (WW-F) | 0.26 AB | 0.06 AB | 0.33 B | 0.59 B | 3.58 AB |
| 10 to 15 cm | WW (WW-C-F) | 0.29 A | 0.08 A | 0.44 A | 0.79 A | 4.66 A |
|  | C (WW-C-F) | 0.18 AB | 0.04 A | 0.29 A | 0.45 AB | 2.91 BC |
|  | F (WW-C-F) | 0.16 B | 0.04 A | 0.24 A | 0.42 B | 2.58 C |
|  | W (WW-F) | 0.29 A | 0.07 A | 0.43 A | 0.77 AB | 4.46 AB |
|  | F (WW-F) | 0.19 AB | 0.04 A | 0.37 A | 0.61 AB | 3.45 ABC |
